# Toward high sustainability using fully recycled geopolymer concrete: mechanical, rheological, and microstructural properties†

**DOI:** 10.1039/d5ra02249e

**Published:** 2025-07-04

**Authors:** Faramarz Moodi, Mohammad Reza Hanafi, Zahra Shariatinia

**Affiliations:** a Department of Civil and Environmental Engineering, Amirkabir University of Technology Tehran Iran fmoodi@aut.ac.ir; b Department of Chemistry, Amirkabir University of Technology Tehran Iran shariati@aut.ac.ir

## Abstract

This study explores the sustainable production of fully recycled geopolymer concrete using waste ordinary Portland cement (OPC) concrete, aiming to reduce environmental impact and promote circular construction practices. Crushed OPC concrete was utilized as both recycled aggregate (RA) and recycled concrete powder (RCP), while recycled clay brick powder (RBP) was incorporated as a supplementary aluminosilicate additive. Alkaline activation was performed using sodium hydroxide solutions at three molarities (8, 12, and 16 M) combined with water glass (WG) at a WG/NaOH ratio of 2. The mechanical, rheological, and microstructural properties of geopolymer recycled concrete (GRC) and paste (GRP) were assessed alongside a Life Cycle Assessment (LCA). Results showed that incorporating RA and RCP significantly enhanced compressive strength—up to 90% at 90 days compared to geopolymer paste—while increasing NaOH molarity improved overall mechanical performance. Although RBP decreased compressive strength, density, and elastic modulus, it notably enhanced workability, surface quality, and crack resistance. Microstructural analysis revealed strong adhesion between RCP and RA due to their chemical compatibility, contributing to matrix homogeneity. The LCA confirmed that GRC exhibits a lower environmental footprint than conventional OPC concrete. These findings support the classification of GRC as a high-performance structural material and demonstrate its potential as a viable, eco-friendly alternative for sustainable construction applications.

## Abstract 2

This study explores the sustainable production of fully recycled geopolymer concrete using waste ordinary Portland cement (OPC) concrete, aiming to reduce environmental impact and promote circular construction practices.

## Graphical abstract

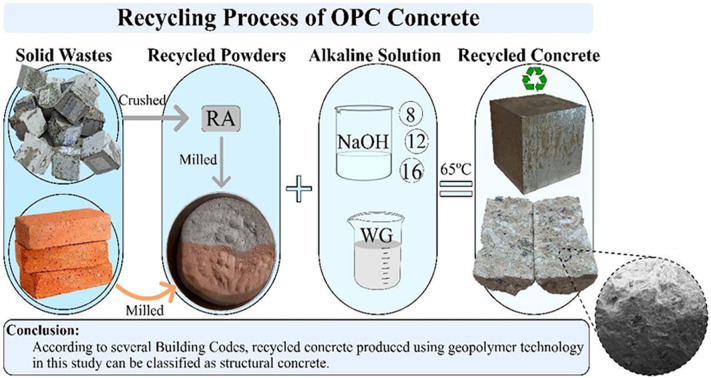

## 1. Introduction

Concrete is one of the most popular and widely used construction materials in the world. Its affordability, strength, and durability under various environmental conditions have made it a sustainable building material in the construction industry.^1–6^ The raw material of concrete is easily accessible, resulting in a simple and economical manufacturing process. However, the cement industry is responsible for around 7% of annual CO_2_ emissions worldwide. Thus, about 4 GJ of energy is consumed to produce each ton of ordinary Portland cement (OPC),^7^ accounting for 12–15% of annual energy used by all industrial sectors worldwide. Moreover, to produce one ton of OPC, about one ton of CO_2_ is released into the atmosphere, and about 60–130 kg of liquid or similar fuel is consumed, and 110 kWh of electricity is needed to create a temperature above 1000 °C for calcination and clinker formation.^8,9^

Due to high energy consumption in cement production processes and the non-recyclability of OPC concrete, construction and demolition waste (CDW) made of concrete must be thrown away and buried or dumped in the environment.^10,11^ Generally, CDW is a type of solid waste that includes a wide range of waste such as concrete, brick, asphalt, wood and plastic generated during construction, rehabilitation or demolition.^12–15^ Growth in urban population and advancement of construction have also led to an increase in demolition of buildings and other structures, which has led to an increase in generation of waste bricks and concrete.^16,17^ Currently, CDWs account for 30–35% of the world's solid wastes,^18–20^ of which 80% are concrete and bricks.^21,22^ Accordingly, recycling these materials has drawn global attention. The second-most widely used building material after concrete is brick, which is still used by many architects and designers in construction.^23,24^ In addition to being relatively easy to obtain, bricks are available in a greater variety of types, shapes and efficiency levels.^25–27^ Consequently, brick waste, like other CDWs such as concrete, has harmful effects on the environment. Furthermore, the majority of these materials can be recycled by incorporating better materials.^28–31^ In fact, recycling of construction waste not only protects natural and ecological resources, but also has economic justification.^32,33^ This reduces the amount of CO_2_ production as well as energy consumption.^34–36^

In line with sustainable development, it is essential to meet the needs of the current generation while trying to protect the needs of future generations.^37–39^ On the other hand, the growth of the industry and the trend towards industrialization require an in-depth study in the field of CDW consumption in order to reduce future challenges. In this regard, recycling and reuse of CDW reduces large amounts of CO_2_ emissions and energy consumption.^34–36^ Consequently, scientists are trying to replace environmentally harmful materials with environmentally friendly ones in order to lessen hazards associated with OPC manufacturing. One such material is geopolymer, which frequently utilizes waste from various industries. With the goal of reducing the negative impact of OPC on the environment, geopolymer binders have received the most research attention among non-cementitious binders.^40,41^ A source of geopolymer binders can generally be any silicon- and aluminum-containing material that interacts in alkaline environment to form polymer chains and interconnected networks.^42–44^ Therefore, pozzolans (active aluminosilicate sources) and alkaline minerals are combined to create geopolymer binders.

Given that production of one ton of OPC emits about 0.8 ton of CO_2_ and releases about 2–3 billion tons of CO_2_ into the atmosphere annually,^5,45–47^ switching from OPC concrete to geopolymer concrete can reduce CO_2_ emissions by about 80–90%.^48–52^ Furthermore, manufacturing aggregates, which always precedes production of concrete, results in additional adverse environmental impacts.^53–55^ Mining and sand-washing processes require a lot of energy and release a considerable amount of waste into the environment. In many regions, scarcity of natural resources for production of construction materials also necessitates their transport from distant locations to the project site.^56,57^ Consequently, transportation costs are added to production costs, increasing the overall expense.^58^ Thus, energy losses and environmental pollution can be avoided by recycling of aggregates and switching to geopolymer concrete in place of OPC concrete. Also, geopolymer concrete has attracted attention due to its early and high compressive strength as well as resistance to fire and aggressive environment and low permeability.^43,45,59–64^ Geopolymer is a suitable material for lightweight concrete and lightweight aggregates can be used instead of regular aggregates.

Compared to OPC concrete, production of GGBFS-based geopolymer concrete typically uses around 40% less energy. Besides, 94% of the total energy consumption for OPC concrete production goes towards OPC itself. However, water glass (WG) accounts for 49% of total energy consumption for production of GGBFS-based geopolymer concrete and NaOH for 39%.^65^ Unlike OPC concrete, where the only variable component is amount of cement, which directly affects its strength and leaves little room for change, geopolymer concrete has two influential components (two alkaline solutions) that provide possibility of different properties to make changes in energy consumption and costs.^66,67^ Furthermore, production of new materials is always expensive and energy-intensive, therefore production of new aggregates for OPC concrete in river sand mining factories is environmentally harmful.^53,68^ Notably, the process used to produce fine and coarse recycled aggregates (RA) in this study is comparable to that used to produce natural aggregates sold on market, with the exception that environmental risks associated with river sand mining have been eliminated in RA. In addition, because the Jaw Crusher and Hammer Mill used in this research are portable and can be transported to project site, the cost of transporting materials from factory to project site is lower.

In addition to the aforementioned instances, about 8 million tons of brick and concrete waste, which require disposal, are generated annually worldwide.^21,22,69^ This creates new materials that consume a lot of resources.^70,71^ In addition, these wastes are usually collected and buried or dumped in environment, which has been criticized for two reasons: it is harmful to environment and consumes resources.^72^ In other words, authors contend that disposal of concrete and brick waste is a waste of resources and money. Because through recycling, these waste products can be returned to the economy and the production of OPC concrete and natural aggregates can be prevented.

The closest attempts to recycle OPC concrete include using RA from crushed OPC concrete in other concretes^30,34,35,73–76^ or pulverizing OPC concrete, increasing the reactivity of the resulting powder by grinding it to very fine particles, and then replacing parts of the OPC with very fine waste concrete powder.^20,36,77–85^ In addition, studies have been conducted on the full recycling of OPC concrete, in which recycled concrete powder (RCP) was rehydrated using standard methods, including intense heating in a cement kiln, re-clinkering, and then mixing with RA to produce recycled concrete.^86–88^ This approach uses a lot of energy to generate intense heat in the kiln and release CO_2_ into the atmosphere, much like it does during the production of OPC.^89,90^ The main drawbacks of this method are its instability, high cost, and excessive energy consumption. Moreover, recycled brick powder (RBP) has been used to prepare geopolymer samples in a number of studies, supporting the great potential of geopolymer in recycling of construction waste.^91–97^

While the experimental procedures and mix designs presented in this study were standardized to ensure repeatability, it is important to acknowledge that the performance of geopolymer recycled concrete (GRC) may be affected by regional differences in the quality and composition of construction and demolition waste (CDW). Variations in the type of cement, degree of hydration, and mineral composition of recycled concrete powder (RCP) and recycled brick powder (RBP) can influence the geopolymerization process and resulting mechanical properties. To enhance reproducibility, this study employed carefully characterized waste materials, with their chemical compositions and phases confirmed through EDS and XRD analysis. Moreover, all specimens were prepared under controlled activation conditions, including fixed WG/NaOH ratios, selected NaOH molarities, and a consistent thermal curing protocol. Although these conditions help ensure methodological consistency, future research is encouraged to validate the presented findings using locally sourced recycled materials to account for contextual variability and confirm broader applicability.

This study addresses a critical research gap in the full recycling of ordinary Portland cement (OPC) concrete without relying on energy-intensive reprocessing methods such as re-clinkering. By simultaneously utilizing crushed OPC concrete as both recycled aggregate (RA) and recycled cementitious powder (RCP), and incorporating recycled brick powder (RBP) as a supplementary source of aluminosilicates, the research introduces a novel geopolymer formulation. Distinguishing itself through several key innovations, the study employs geopolymerization to transform OPC waste into a fully recycled concrete product, thereby avoiding the environmental burden of conventional recycling processes. It systematically investigates the influence of varying sodium hydroxide (NaOH) molarities and water glass/NaOH ratios on the performance of the geopolymer recycled concrete (GRC), optimizing the alkaline activation process for OPC waste materials. Through comprehensive evaluations of mechanical, rheological, microstructural, and sustainability aspects—including microstructural analysis confirming effective paste-aggregate adhesion and a detailed life cycle assessment (LCA) quantifying environmental benefits—the study demonstrates that the developed GRC not only meets structural performance standards but also offers a viable, low-carbon alternative for sustainable construction practices.

## 2. Material and methods

### 2.1. Materials

In this study, for the first time in the production of concrete, the consumption of OPC cement and natural aggregates was reduced to zero, and by crushing and grinding waste OPC concrete (compressive strength of 30–50 MPa), both RCP and RA were provided for production of GRC and GRP. Also, RBP was used as an additive to control and change some properties of GRC. Additionally, the energy dispersive Energy Dispersive X-ray Spectroscopy (EDS) test was performed on RCP and RBP to analyze the chemical elements. The results are shown in Table 1.

**Table 1:** Chemical components of RCP and RBP

	Wt%										Atom			
	O	Na	Mg	Al	Si	K	Ca	Ti	Mn	Fe	Si/Al	Na/Si	Na/Al	Ca/Si
RBP	53.31	1.36	2.82	7.71	21.97	1.11	7.78	0.28	0.35	3.3	2.74	0.08	0.21	0.25
RCP	54.39	0.92	1.08	5.81	20.21	2.22	12.6	0.23	0.33	2.24	3.34	0.06	0.19	0.44

Due to the high concentration of calcium, silicon and aluminum in RCP, it is a suitable source of aluminosilicate and can be used to produce geopolymers. In addition, water glass (WG) with a ratio of SiO_2_/Na_2_O:2.5 and industrial sodium hydroxide (NaOH) with a purity of 98% were used as alkaline activator.

**Fig. 1:**
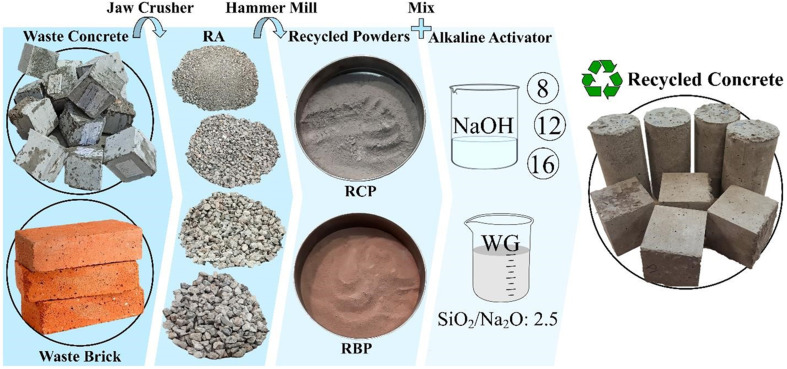
Recycling process of OPC concrete into geopolymer recycled concrete (GRC), including material crushing, powder production (RCP, RBP), and mixing with alkaline activators (NaOH, WG) to form recycled concrete specimens.

It has been attempted to prepare waste concrete from different sites in order to bring the laboratory conditions closer to actual concrete recycling circumstances. This is because the randomness of the types of waste concrete and the variety of the cement types used in the waste concrete are uncontrollable parameters. The sole controllable variable is the minimum compressive strength of waste concrete, which is directly related to the RA strength derived from waste concrete. In other words, once the concrete recycling plan is operational, controlling the quality of the concrete and the type and amount of cement used is impossible. The concrete that is collected for recycling is evident that it is a part of the structure's column, beam, and slab, or other elements of the building. It is crucial for the accuracy of the research's findings that both the RA and the RCP are derived from waste concretes with a minimum compressive strength of 20 MPa. It is important to note that the production of OPC concrete in the laboratory and the extraction of RA from it was avoided. As shown in Fig. 1, cubic waste OPC concretes were crushed with a jaw crusher to obtain RA, and the crushed materials were then granulated by passing through different sieves. The papers pertinent to this topic were used in order to acquire the proper granulation^98–101^ and finally, after numerous trials, the best granulation was achieved. The density and particle size of the aggregates obtained are listed in Table 2. Then, after being crushed by a jaw crusher, a portion of the OPC concrete and brick was ground into powder with a hammer mill so that it could be utilized as a binder in GRC. Thereafter, RCP and RBP were obtained by passing the recycled powders through sieve no. 200 (75 μm).

**Table 2:** Mix design of geopolymer specimens (kg m^−3^)

	Specimen	RCP	RBP	NaOH	WG	l/b^a^	Coarse RA (sieve)			Fine RA (sieve)
							1–3/4	3/4–1/2	1/2–4	4–50
Concrete	C-100C-0B-8-2	570	0	85.5	171	0.45	170	280	510	470
	C-100C-0B-12-2	570	0	85.5	171	0.45	170	280	510	470
	C-100C-0B-16-2^b^	570	0	85.5	171	0.45	170	280	510	470
	C-95C-5B-8-2	541.5	28.5	85.5	171	0.45	170	280	510	470
	C-95C-5B-12-2	541.5	28.5	85.5	171	0.45	170	280	510	470
	C-95C-5B-16-2^c^	541.5	28.5	85.5	171	0.45	170	280	510	470
	C-90C-10B-8-2	513	57	85.5	171	0.45	170	280	510	470
	C-90C-10B-12-2	513	57	85.5	171	0.45	170	280	510	470
	C-90C-10B-16-2	513	57	85.5	171	0.45	170	280	510	470
	C-85C-15B-8-2	484.5	85.5	85.5	171	0.45	170	280	510	470
	C-85C-15B-12-2	484.5	85.5	85.5	171	0.45	170	280	510	470
	C-85C-15B-16-2	484.5	85.5	85.5	171	0.45	170	280	510	470
Paste	P-100C-0B-8-1	1600	0	320	320	0.4	—	—	—	—
	P-100C-0B-8-2	1600	0	213	427	0.4	—	—	—	—
	P-100C-0B-12-1	1600	0	320	320	0.4	—	—	—	—
	P-100C-0B-12-2	1600	0	213	427	0.4	—	—	—	—
	P-100C-0B-16-1^d^	1600	0	320	320	0.4	—	—	—	—
	P-100C-0B-16-2	1600	0	213	427	0.4	—	—	—	—
	P-95C-5B-8-1	1520	80	320	320	0.4	—	—	—	—
	P-95C-5B-8-2	1520	80	213	427	0.4	—	—	—	—
	P-95C-5B-12-1	1520	80	320	320	0.4	—	—	—	—
	P-95C-5B-12-2	1520	80	213	427	0.4	—	—	—	—
	P-95C-5B-16-1^e^	1520	80	320	320	0.4	—	—	—	—
	P-95C-5B-16-2	1520	80	213	427	0.4	—	—	—	—
	P-90C-10B-8-1	1440	160	320	320	0.4	—	—	—	—
	P-90C-10B-8-2	1440	160	213	427	0.4	—	—	—	—
	P-90C-10B-12-1	1440	160	320	320	0.4	—	—	—	—
	P-90C-10B-12-2	1440	160	213	427	0.4	—	—	—	—
	P-90C-10B-16-1	1440	160	320	320	0.4	—	—	—	—
	P-90C-10B-16-2	1440	160	213	427	0.4	—	—	—	—
	P-85C-15B-8-1	1360	240	320	320	0.4	—	—	—	—
	P-85C-15B-8-2	1360	240	213	427	0.4	—	—	—	—
	P-85C-15B-12-1	1360	240	320	320	0.4	—	—	—	—
	P-85C-15B-12-2	1360	240	213	427	0.4	—	—	—	—
	P-85C-15B-16-1	1360	240	320	320	0.4	—	—	—	—
	P-85C-15B-16-2	1360	240	213	427	0.4	—	—	—	—

a l/b (liquid to binder): ratio of alkaline solution to recycled powders.

b C-100C-0B-16-2 represents specimen made of 100% RCP and 0% RBP at 16 M NaOH concentration and R2 (WG/NaOH:2).

c C-95C-5B-16-2 represents specimen made of blend mix of 95% RCP and 5% RBP at 16 M NaOH concentration and R2.

d P-100C-0B-16-1 represents specimen made of 100% RCP and 0% RBP at 16 M NaOH concentration and R1 (WG/NaOH:1).

e P-95C-5B-16-1 represents specimen made of blend mix of 95% RCP and 5% RBP at 16 M NaOH concentration and R1.

Considering that in geopolymers, NaOH solution is used as a means of decomposing the aluminosilicate source and converting it into its oxide components;^102,103^ in this study, NaOH concentrations of 8, 12, and 16 M and WG/NaOH:1 & 2 (R1 & R2) were used to prepare geopolymer samples. Because of sodium's large ionic radius and its low attraction for its OH^−^ anion, it readily breaks down into its individual ions (Na^+^ and OH^−^) in water. Due to the strong solubility property of the NaOH solution, most aluminosilicate sources are dissolved in these solutions and are therefore often used in the field of geopolymers.^104^

In the case of WG, the higher the concentration of SiO_2_/Na_2_O, the more viscous the WG solution. Viscosity is a product of the formation of silicate polymers from Si and O atoms in WG. These atoms are linked by covalent bonds into large negatively charged chains or ring structures that attract positively charged Na^+^ and H_2_O molecules.^105^ WG contributes to the formation of an initial silica gel upon activation of the geopolymer binder. This gel is formed when soluble silicates react with Ca^2+^ ions in the binder to form CASH or in some cases CSH. In addition, SiO_2_ in WG also improves the strength gain of geopolymer concrete.^106^

### 2.2. Experimental methods

In the preparation of geopolymer samples, RCP was used as the binder and RBP at 5, 10, and 15 wt% as the additive for placement instead of RCP. The alkaline solution was prepared in advance and the required amount was weighed into a glass container. Thereafter, RCP and RBP were weighed and mixed in an electric paste mixer to obtain a homogeneous mixture.^107^ Then, the alkaline solution was gradually added to the binder and mixed for 5 minutes. In the case of concrete preparation, at this stage, the aggregates were weighed and mixed by a concrete mixer so that the granulation of the aggregates was almost the same in all places. Then, the geopolymer paste was gradually added and mixed for 5 minutes. Finally, the prepared geopolymer paste was poured into paste molds measuring 5 × 5 × 5 cm, 4 × 4 × 16 cm, and briquette (8-shape) and concrete molds measuring 10 × 10 × 10 cm (cubic), 10 × 10 × 50 cm (primastic) and 10 × 20 cm (cylindrical), and these molds were then placed on the vibrating table for 30 seconds to fully condense. It should be noted that the geopolymer paste was self-compacting and once poured into the mold, it immediately flowed in all directions, filling every gap. After molding, the samples were placed in a 65 °C oven for two hours. The molds were then removed and the samples cured for three days before being stored at ambient temperature until the day of evaluation. To ensure the reproducibility and reliability of the results, three replicate specimens of both concrete and mortar were prepared for each mix design and tests. These specimens were then subjected to compressive, flexural, and tensile strength tests, as well as water absorption tests. Table S2† shows the mix design for GRC and GRP samples.

Mechanical strength tests were carried out on paste and concrete samples aged 3, 7, 28 and 90 days. In addition, the compressive strength was measured using a hydraulic compression testing machine that conformed to ASTM-C39/C39M-23 standards.^108^ Based on the ASTM-C348-21 standard,^109^ the three-point loading method (exerting a concentrated force on the beam with two supports) was used to measure the flexural strength of the paste and concrete samples. For this purpose, metal prism molds measuring 4 × 4 × 16 cm and 10 × 10 × 50 cm were used and the flexural strength of the paste and concrete samples was assessed at the age of 3, 7, 28 and 90 days. For tensile strength, briquette specimens were used to measure the tensile strength of the paste according to ASTM-C190:1985 (ref. 110) and 10 × 20 cm cylindrical specimens according to ASTM-C496-96 (ref. 111) were used to evaluate GRC and GRP splitting tensile strength. Additionally, the SEM for microstructure determination was performed on the fractured surface of cylindrical GRC samples. For this purpose, the MIRA3 FEG-SEM machines, manufactured by TESCAN in the Czech Republic, were used. It features a field emission film and operates in both high vacuum and low vacuum modes (ideal for non-conductive materials); and can magnify objects up to a million times with the voltage of 30 kV and a resolution of up to 1 nm. This machine was equipped with an EDS detector and was used to detect the elements in RBP and RCP. Additionally, chemical and microstructural characterization was achieved through X-ray fluorescence (XRF) (Table 2). Also, RBP, RCP, and GRC (C-100C-0B-16-2) underwent XRD analysis. The aforementioned XRD machine was a Chinese-made TD-3700 type with a copper-based X-ray lamp anode and a Kα_1_ copper-radiation X-ray source, in which the wavelength is 1.5406 Å. The analysis accuracy of the device is 0.02° per 0.5 seconds, and the voltage and current employed are 30 kV and 20 mA, respectively. It evaluated the samples with 2*θ* angles between 10° and 70°. Additionally, workability and flowability of the concrete were evaluated using the slump test in accordance with ASTM-C143/C143M-12 standard.^112^ Regarding the paste, the flow test was carried out using the flow table according to the ASTM-C1437-20 standard.^113^ Additionally, the water absorption test and density evaluation of the GRC and GRP samples have been conducted in accordance with the ASTM-C1585-20 standard.^114^ The elasticity modulus was assessed using fifteen cylindrical concrete specimens measuring 20 × 10 cm, in compliance with the ASTM-C469/C469M-14 standard.^115^ This step ensures that properties of GRC and GRP align with recognized industry benchmarks, contributing to their viability as sustainable building materials.

Finally, a comprehensive life cycle assessment (LCA) was conducted on the bio-composite samples in accordance with ISO 14040:2006 (ref. 116) and ISO 14044:2020 (ref. 117) standards to quantify their environmental impacts.

## 3. Results and discussion

This study explores the sustainable production of fully recycled geopolymer concrete using waste ordinary Portland cement (OPC) concrete, aiming to reduce environmental impact and promote circular construction practices. Crushed OPC concrete was utilized as both recycled aggregate (RA) and recycled concrete powder (RCP), while recycled clay brick powder (RBP) was incorporated as a supplementary aluminosilicate additive. Alkaline activation was performed using sodium hydroxide solutions at three molarities (8, 12, and 16 M) combined with water glass (WG) at a WG/NaOH ratio of 2. The mechanical, rheological, and microstructural properties of geopolymer recycled concrete (GRC) and paste (GRP) were assessed alongside a Life Cycle Assessment (LCA). This section showed the presentation of the results in a detailed way.

### 3.1. Mechanical strength of paste

This section evaluates how the mechanical properties of geopolymer paste vary with changes in RCP content, RBP substitution levels, NaOH molarity, and WG/NaOH ratio. Results are presented in Fig. 1a–c, showing compressive, tensile, and flexural strength trends across curing ages. These variations are interpreted in the context of gel structure evolution and binder composition. Recycled concrete is a valuable resource for construction industry, attained by demolishing and reusing old concrete structures, offering both economic and environmental benefits. This section investigates the mechanical strength of recycled pastes with the main goal of identifying paste with the highest mechanical strength and the most efficient combination of components. Additionally, this study examines how various parameters affect paste's behavior. Table 2 presents chemical components of RCP and RBP. Fig. 1 reveals processes performed for OPC concrete recycling and conversion to GRC. Also, Table 2 indicates mix design of geopolymer specimens (kg m^−3^).

**Fig. 2:**
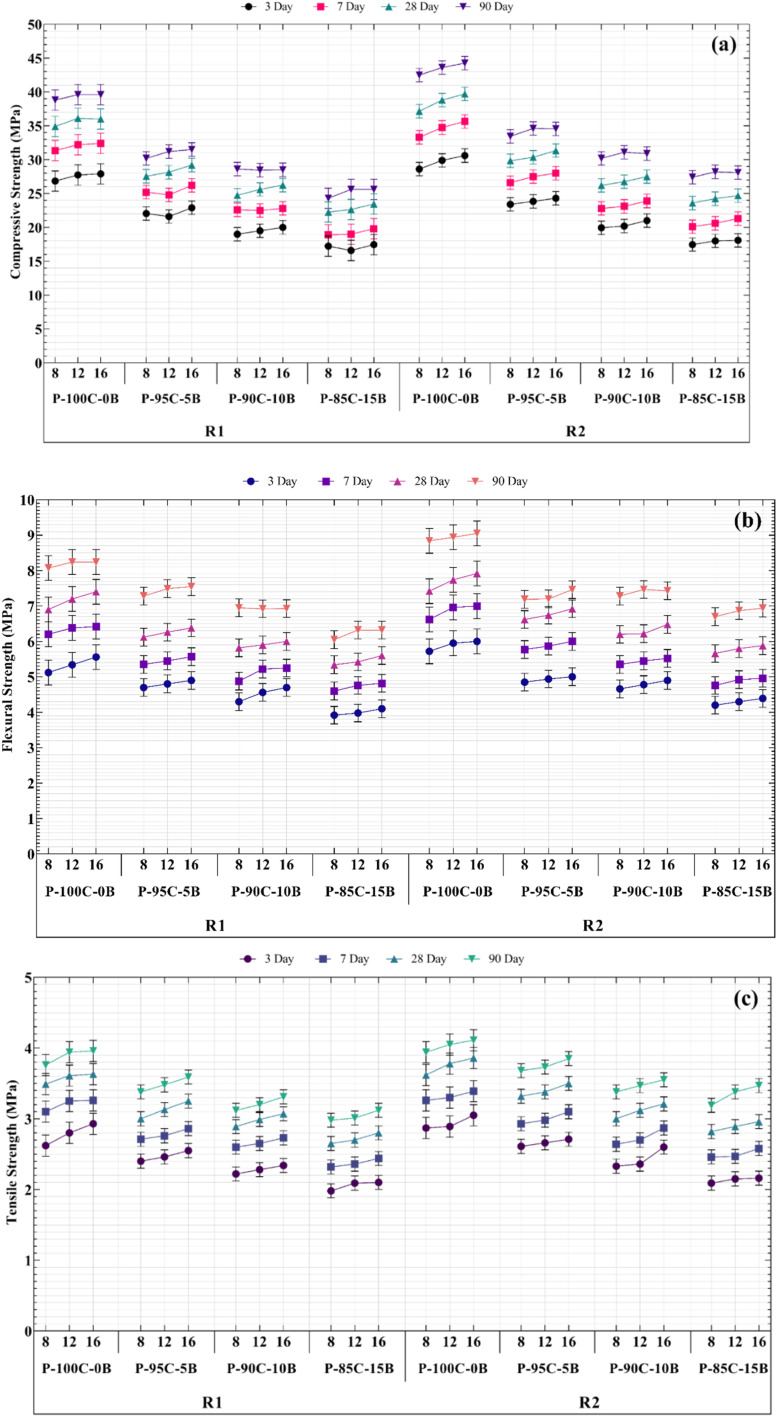
Mechanical performance of GRC samples at different curing ages: (a) compressive strength, (b) flexural strength, and (c) splitting tensile strength, showing the effect of varying NaOH morality and WG/NaOH rations. Error bars represent standard deviations from triplicate tests.

Fig. 2 depicts variations in geopolymer paste's mechanical strength, which illustrates that as RCP values of samples increase, so does its mechanical strength. In addition, as R (WG/NaOH) increases, the mechanical strength also increases. As a result, 90 days compressive strength in P-100C-0B-16-1 and P-100C-0B-16-2 pastes is 39.60 and 44.25 MPa, respectively, an increase of about 12%. This number for flexural strength reaches 8.24 and 9.05 MPa (an increase of about 10%) and tensile strength also reaches 3.96 and 4.11 MPa, which shows an increase of about 4%. These results are consistent with previous studies according to which WG in geopolymers is used as a stiffening agent in geopolymer structure and increasing its amount in paste samples leads to an increase in hardness and an increase in mechanical strength.^3,118,119^ Furthermore, increasing NaOH concentration affects mechanical strength of geopolymer paste.

Accordingly, increasing NaOH concentration from 8 to 12 and 16 M in P-100C-0B-8-2, P-100C-0B-12-2 and P-100C-0B-16-2 pastes results in an increase in 90 days compressive strength of 42.5 to 43.6 and 44.25 MPa, respectively, which represents increases of about 2.6% and 4.1%. The corresponding flexural strength values are 8.84 to 8.94 and 9.05 MPa, an increase of about 1.1% and 2.4%, respectively, and tensile strength showed an increase of 2.8% and 4.3%, respectively. When comparing percentage changes in compressive strength and flexural strength of geopolymer paste samples caused by variations in the molarities of NaOH, it is found that these percentage changes are almost identical. In addition, mechanical strength of the geopolymer paste was significantly reduced by substituting 5, 10 and 15 wt% of RBP for RCP. Therefore, in the 90 days samples of P-100C-0B-16-1 and P-100C-0B-16-2, by adding only 5 wt% RBP, the compressive strength reached from 39.6 to 31.5 MPa and from 44.25 to 34.55 MPa, respectively. It shows a decrease of about 25.7% and 28.1%. This decrease is about 9.1 and 21.3% for flexural strength and for tensile strength is 10.3% and 6.8%. Also, impact of increasing RBP content on compressive strength in samples of 10B-16-1 and 15B-16-1 compared to free-RBP samples is equal to 38.9% and 54.7%, for flexural strength is equal to 18.6% and 30.4%, and for tensile strength is equal to 19.6% and 26.9%, respective.

Likewise, for R1 and R2 samples in all types of paste, amount of mechanical strength loss brought on by the addition of RBP was approximately the same scale. Additionally, the same mechanical strength values among all samples from 3, 7, 28, and 90 days indicated that geopolymer recycled paste (GRP) samples rapidly gain strength at early ages, which is a result of 3 days curing at 65 °C in oven.

### 3.2. Mechanical strength of concrete

**Fig. 3:**
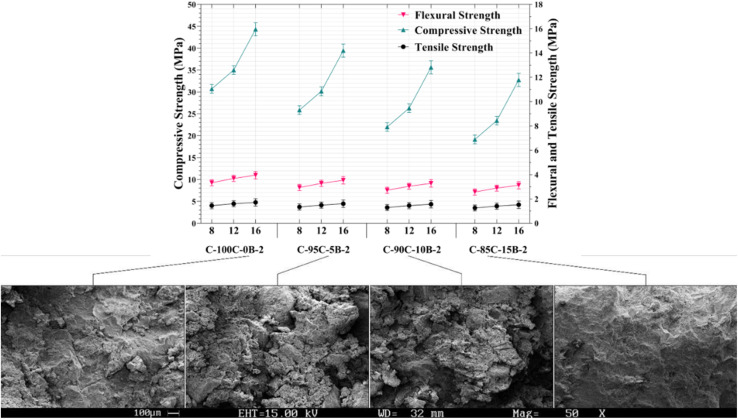
Compressive and splitting tensile strength of 28 days GRC mixtures incorporating RBF, along with corresponding SEM micrographs at 50× magnification. The SEM images illustrate the microstructural variations associated with strength differences.

Building on the paste data, this section presents the compressive and tensile strength behavior of geopolymer recycled concrete (GRC). Comparisons are made between RBF-free and RBF-substituted concretes, and strength values are benchmarked against international structural classification limits. SEM images support the interpretation of strength trends. Considering that the X-X-2 samples had the highest compressive strength in the paste. Therefore, a concrete compressive strength test for an alkaline solution containing WG/NaOH:2 and NaOH with a concentration of 8, 12, and 16 M was designed and carried out. Meanwhile, the use of different amounts of RBP as a variable additive and its impact on compressive strength of geopolymer concrete has been studied. Similar to the mechanical strength of paste, the 28 days compressive strength of GRC samples decreased by about 21% when 5 wt% RBP was substituted for RCP. Furthermore, compared to GRC with free-RBP, adding 10 and 15 wt% of RBP reduced the compressive strength by 30% and 37.5%, respectively. Thus, use of RA in GRC did not affect the rate at which compressive strength decreased as a result of the addition of RBP, and this decrease was consistent with the paste findings. Likewise, comparing Fig. 2 and 3 shows that for 28 days samples of C-100C-0B-16-2, C-95C-5B-16-2, C-90C-10B-16-2 and C-85C-15B-16-2 the compressive strengths of paste and GRC varies by 4.6, 3.65, 3.2 and 2.95 MPa respectively, or an average of 11.7%.

The corresponding values for the addition of 5, 10, and 15 wt% of RBP in 28 days were 34.4, 42, and 25.4%, respectively. As a result, even a small addition of RBP (5 wt%) resulted in a rapid drop in splitting tensile strength, and as RBP addition increased, this rate decreased until it eventually tended to zero. Previous studies have indicated that tensile strength and compressive strength of concrete are closely related, which is demonstrated using non-linear equations.^120^ Therefore, it is evident from Fig. 3 that the 28 days compressive strength of geopolymer concrete is approximately 10.46% of the corresponding compressive strength. In addition to foregoing, the GRC developed in this research is considered to be a structural concrete according to the European concrete standards (EN 934) and the ACI (minimum compressive strength: 20 MPa) and national building regulations of Iran (minimum compressive strength: 25 MPa).

SEM images in Fig. 3 exhibit that microstructure of GRC is influenced by RBP addition. In C-100C-0B-16-2 sample without RBP, the geopolymer gel particles exhibit uniform distribution and strong connectivity, while aggregate particles are fully surrounded by the gel matrix. This uniform structure indicates high strength and durability. However, in C-95C-5B-16-2 sample (with 5% RBP), irregularly distributed RBP particles lead to voids and cracks, compromising strength and increasing susceptibility to water infiltration and chemical damage. C-90C-10B-16-2 sample (with 10% RBP) shows more pronounced voids and cracks, further weakening the concrete. Finally, C-85C-15B-16-2 sample (with 15% RBP) exhibits significantly abundant voids and cracks, severely compromising structural integrity. Overall, caution is advised when incorporating RBP into GRC and GRP due to its impact on microstructural properties and concrete performance.

**Fig. 4:**
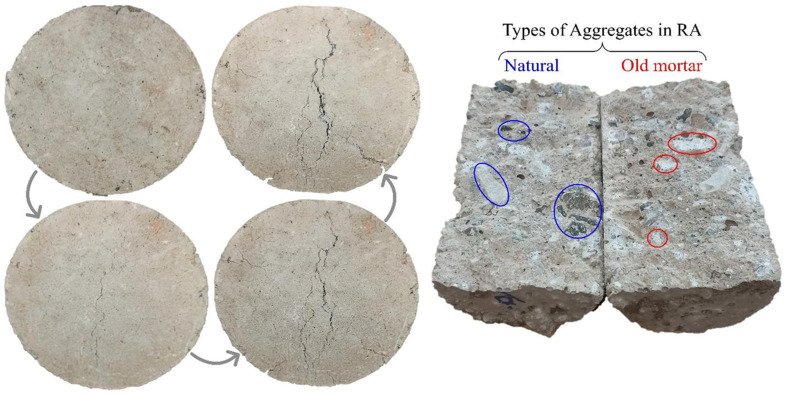
(Left) Surface cracking and fracture propagation in GRC specimens after drying cycles. (Right) Visual identification of aggregate types in RA, highlighting natural aggregates (blue) and adhered old mortar (red).

Splitting tensile strength test was used to study the fractures developed in the concrete to assess the manner in which the particles adhered in the GRC. Generally, when a tensile force is applied to a cylindrical concrete specimen, internal stresses are experienced, causing the material to deform and eventually crack. In the splitting tensile strength test, diagonal compressive stresses are applied to the two opposing ends of concrete specimens until failure occurs along vertical plane passing through center of cylinder. This stress distribution helps explain the development of fractures in concrete under splitting tensile forces. When first microcracks begin, they have the potential to spread or extend deeper into the concrete due to the applied tensile forces. Cracks frequently travel the least impeded paths, such as through weak planes in the concrete matrix or at boundaries between aggregate particles. These microcracks expand and coalesce as further force is applied to create larger, visible fractures that propagate across entire cross-section of specimen until final collapse.^121,122^Fig. 4 illustrates surface of cylinder in GRC's fractured plane in addition to the crack's progression. Recycled aggregate (RA), derived from crushed waste concrete, contains previously used natural aggregates. Given that RCP and concrete-based RA are consubstantial, the alkaline solution used to prepare the GRC samples interacted not only with RCP but also with the surface of the concrete-based RA during the mixing stage, resulting in strong adhesion between them. On the other hand, the fact that RA has sharp edges also contributes to strength of bond between RA and paste. In other words, adhesion between geopolymer paste and concrete-based RA surface overcomes adhesion between natural aggregates and old paste, and when GRC is broken, connection between natural aggregates and old paste will break sooner.

Fig. 4 indicates that there is good adhesion between RCP and RA. This is because RCP and RA are consubstantial, which means that during concrete mixing stage, part of alkaline solution also reacts with the RA surface, creating conditions for strong adhesion between RCP and RA.

**Fig. 5:**
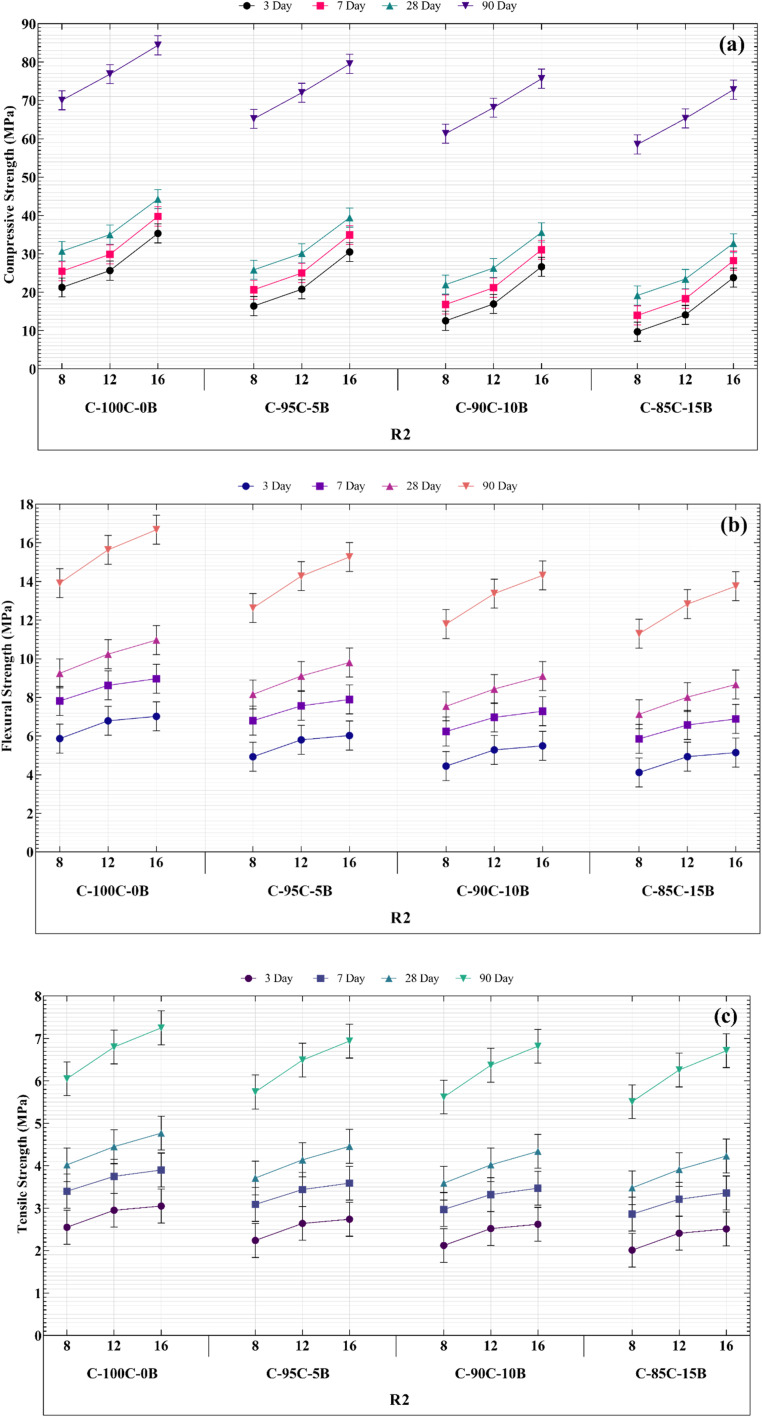
Mechanical strength of GRC mixtures incorporating RBP and RCP at 7 and 28 days: (a) compressive strength, (b) flexural strength, and (c) splitting tensile strength. Results highlight the influence of recycled powder type and curing time on performance development.

Alongside geopolymer paste samples, geopolymer concrete samples aged 3, 7, 28, and 90 days were fabricated and assessed for mechanical strength (Fig. 5). As previously discussed, adding RBP notably diminishes the compressive, flexural, and tensile strengths of GRC. However, the noteworthy increase in GRC's mechanical strength at 90 days is significant, with the highest compressive strength of 90 days GRC showing 190% increase compared to the highest strength of 28 days-old GRC. Similarly, sample C-100C-0B-16-2 exhibited the greatest mechanical strength, with nearly a 152% increase in both flexural and tensile strengths. The primary reason for this is ongoing geopolymerization reaction between the raw materials (aluminosilicates and activating alkalis) in geopolymer concrete. This reaction results in the formation of polymeric gels and siliceous frameworks that bond the aggregate particles, thereby reinforcing concrete structure. Over time, these polymeric gels and siliceous frameworks densify and harden, significantly enhancing the concrete's compressive strength. In the initial stages of geopolymerization, microstructure of geopolymer concrete is relatively rough and porous. As time progresses, polymeric gels and siliceous frameworks refine the microstructure, filling pores and voids, leading to increased density and reduced porosity, which in turn enhances the compressive strength. In geopolymer concrete, various components (polymeric gels, siliceous frameworks, new phases, and aggregate particles) synergize and mutually reinforce each other to increase concrete's compressive strength. Notably, these results are presented for the first time, and the developed GRC falls into the category of High-Performance Concrete (HPC).

The effect of recycled brick powder (RBP) on the mechanical properties of geopolymer recycled concrete (GRC) was systematically evaluated at inclusion levels of 0%, 5%, 10%, and 15% by weight of recycled concrete powder (RCP). The results showed that while the addition of RBP improved workability and visual quality of the concrete, increasing its content led to a consistent reduction in mechanical strength—particularly in compressive and tensile performance. At the 15% substitution level, strength reductions exceeded 30%, indicating a significant compromise in structural integrity. Based on these findings, 15% RBP was considered the upper practical limit under the current mix design and curing conditions. Although higher RBP percentages could be explored in future studies, preliminary results suggest that structural performance would be increasingly impaired. We recommend that future research investigate a broader range of RBP levels across various binder systems and curing regimes to determine application-specific thresholds—especially for differentiating between structural and non-structural concrete applications where workability gains may outweigh strength reductions.

### 3.3. Flowability

Flowability was investigated using slump and flow tests for GRC and GRP, respectively. The section examines the influence of RBF percentage and NaOH molarity on fresh-state behavior. Figures and discussions link flowability to rheological characteristics and the reactive surface interaction between RA and the alkaline solution. Purpose of concrete slump and paste flow tests was to investigate flowability of fresh materials and their ability to change shape under specific stress types. For concrete, only effect of different RBP amounts was examined while effects of all parameters were investigated for paste. It should be mentioned that we had to increase l/b: 0.4 to 0.45 to control concrete flow and improve its workability (Table S1†). Since RA reacts with alkaline solutions, part of the solution was utilized to react with RA's surface. This resulted in a drop in l/b, which significantly reduced flowability and workability and made mixing operations difficult.

**Fig. 6:**
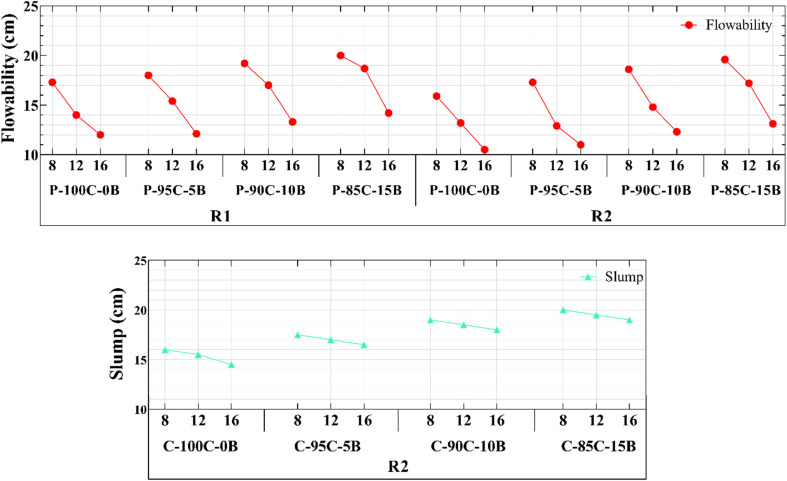
(a) Flow diameter of GRP paste mixtures and (b) slump height of GRC samples with varying activator compositions. Results show decreasing workability with increasing RBP content.

Fig. 6a shows that fluidity of paste decreases as the WG amount in paste increases (from R1 to R2). Additionally, fluidity significantly diminishes with an increase in alkaline solution's concentration. Increasing the NaOH concentration reduced the fluidity of paste for samples P-C8-2 and P-100C-0B-16-2 from 158.5 to 105 mm, a decrease of approximately 33%. Furthermore, including Recycled Brick Powder (RBP) increases the fluidity of both concrete slump and flowability paste. Hence, flowability of paste increased from 105 to 110 mm in P-100C-0B-16-2 by adding only 5 wt% of RBP. Corresponding values for slump in Fig. 6b are increased from 14.5 to 16.5 cm, respectively, which is about 14% of increase. With an increase in RBP from 0% to 10% and 15%, concrete slump exhibited increases of 24% and 34%, respectively. Generally, adding RBP to geopolymer paste and concrete increased the flowability with a linear behavior, which is due to nature of brick. According to EDS results, RBP contains significant amounts of Si, whereas Recycled Concrete Powder (RCP) is primarily composed of Ca and Si. Therefore, Ca has a high reaction rate against alkaline environment and reacts with Si and Al to form CSH and CASH.^60^

### 3.4. SEM/EDS

**Fig. 7:**
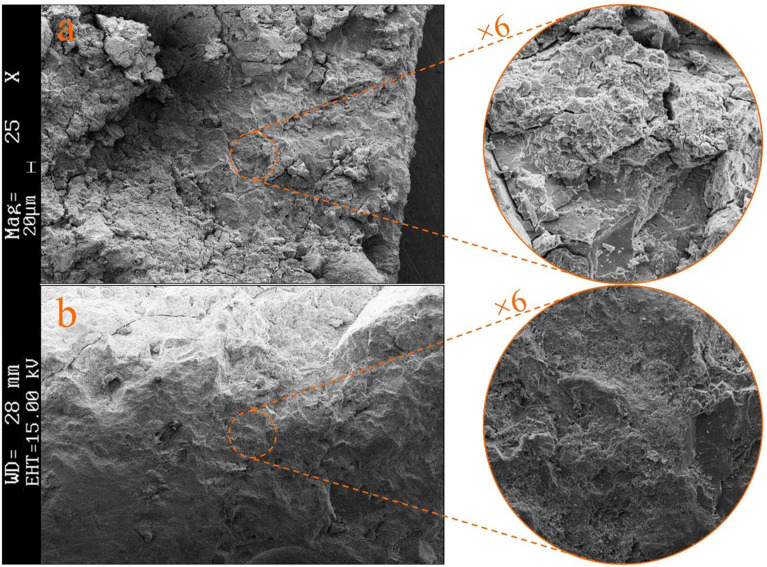
SEM images of GRC samples containing (a) 0 wt% and (b) 15 wt% RBP at 25× magnification, with highlighted regions shown at 150×. The micrographs illustrate changes in matrix density and morphology due to RBP incorporation.

SEM micrographs and EDS spectra illustrate microstructural differences in samples with and without RBF. The discussion interprets changes in gel morphology, void structure, and crack propagation pathways. Strong adhesion between RA and matrix, and homogeneity of the geopolymer phase, are emphasized. Surface of a fractured GRC sample was subjected to SEM analysis to gain insight into microstructure and effects of adding RBP to GRC. Concretes with 0 and 15 wt% RBP are shown in the SEM images of GRC samples in Fig. 7 at 25× and 150× magnifications.

The images reveal that concrete with high RBP content has a more uniform surface and fewer visible cracks. Although Free-RBP concrete has more cracks, it has higher mechanical strength than 15B-16-2 concrete. Additionally, due to high NaOH concentration in alkaline solution, recycled powders have completely decomposed and reacted; as a result, no unreacted components were visible in SEM images. Thus, it is inferred that reduction in mechanical strength due to RBP addition is contingent upon the structure and network formed within the geopolymer.

EDS analysis was performed with the aim of identifying and quantifying the elemental composition of individual phases in the material's matrix.^123,124^ Usually, it is possible to optimize geopolymer concrete and more easily predict its behavior using EDS analysis to understand how elemental composition affects properties and behavior. This allows for development of a more sustainable infrastructure in the development of concrete recycling through geopolymer technology.

**Fig. 8:**
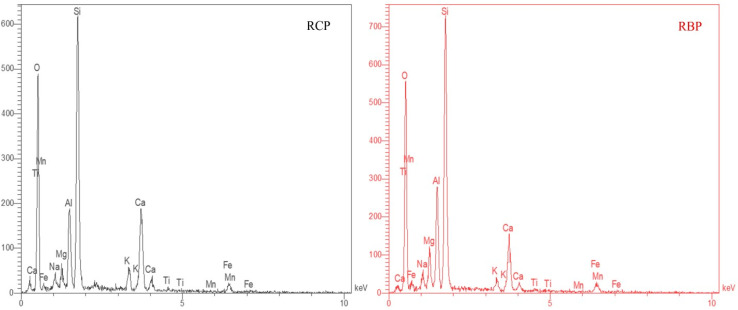
EDS spectra of recycled cementitious powder (RCP, left) and recycled brick powder (RBP, right), showing elemental compositions. Both powders are rich in Si, Al, and O, indicating their potential for geopolymerization.

Ratios of Si/Al, Na/Si, Na/Al, and Ca/Si are presented in Table 2 along with elemental quantities in recycled powders in Fig. 8. Given that (Ca/Si)_RCP_ > (Ca/Si)_RBP_ and (Si/Al)_RCP_ > (Si/Al)_RBP_, it is evident that RCP consists of significant amounts of Ca, which can enhance the mechanical properties of geopolymer concrete. Prior studies have demonstrated that Ca is more reactive than Si and the higher the Ca/Si ratio, the higher the mechanical strength of the geopolymer.^60,125,126^ EDS results in Table 2 and Fig. 8 unveil that RCP has a higher Ca concentration than RBP, and both RBP and RCP are considered suitable sources of aluminosilicate due to their high Si and Al contents. Furthermore, due to (Si/Al)_RCP_ > 3 and (Si/Al)_RBP_ > 2, they have great potential for generating three-dimensional structures.^43,64^ Since Si, Al, and Ca are considered to be fundamental and crucial elements in the manufacture of geopolymers, they make up majority of constituents of these materials.

### 3.5. XRD

XRD results are analyzed to identify crystalline and amorphous phases in RCP, RBP, and GRC. A broad hump in the 20°–35° 2*θ* range indicates semi-amorphous gel phases (N-A-S-H and C-A-S-H), while superimposed sharp peaks (*e.g.*, quartz, C2S) are indexed with JCPDS and numbers and (*hkl*) values. The discussion relates these features to geopolymerization effectiveness and structural stability.

**Fig. 9:**
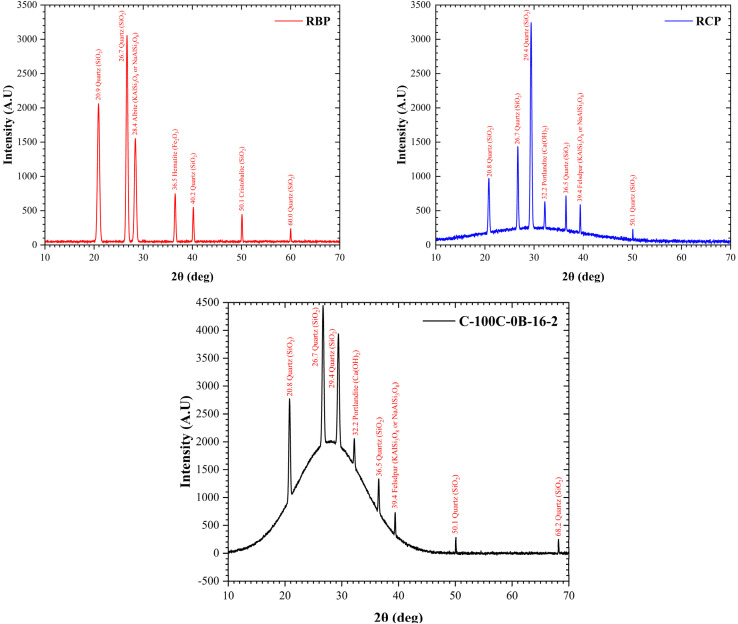
XRD patterns of (left) recycled brick powder (RBP), (middle) recycled cementitious powder (RCP), and (right) geopolymer concrete sample (C-100C-0B-16-2). Major crystalline phases identified include quartz (SiO_2_), albite, hematite, portlandite, and feldspar, with the GRC sample showing a broad hump indicative of amorphous geopolymer gel formation.

XRD analysis was performed to study microstructures of recycled powders and influence of RCP on formation of different phases in curing process and strength of GRC. Overall, XRD analysis provides valuable insights for understanding microstructure–property relationships of innovative materials.^127,128^Fig. 9 shows that RCP peaks are also present in concrete sample, which has undergone slight changes. Therefore, it can be claimed that these changes are due to alkaline reactions and geopolymerization. Considering that RCP is derived from OPC concrete, similar peak values of GRC and RCP indicate the nearly similar structure of these two materials. In addition, due to closeness of Na/Al and Na/Si values, as well as Ca, Si and Al values in RBP and RCP, they were found to have the same crystal structure, with quartz forming the highest peaks (Fig. 9).

It is worth noting that the diffraction pattern of GRC shows both broad hump-like features and sharp crystalline peaks. The broad hump, particularly around 20°–35° 2*θ*, is characteristic of semi-amorphous phases such as N-A-S-H and C-A-S-H gels, which form the primary binding matrix in geopolymer systems. These gel phases are poorly crystalline and indicate the presence of short-range ordering within the aluminosilicate network, rather than long-range crystalline structure. The superimposed sharp peaks correspond to residual or newly formed crystalline phases, including quartz (JCPDS 46-1045), C2S (JCPDS 33-0302), and other mineralogical inclusions. This mixed-phase composition reflects the partial dissolution and transformation of the precursor materials during alkaline activation. The presence of both amorphous and crystalline phases is consistent with prior studies on alkali-activated binders and confirms the formation of a stable yet heterogeneous geopolymer matrix.

This dual-phase behavior has significant implications. The amorphous gel phases contribute to early-age strength development and dense microstructure, while crystalline inclusions influence long-term durability and thermal stability. The presence of a hump in the background does not imply structural instability but instead reflects the intrinsic structural nature of geopolymerization products, as also reported in previous studies of similar alkali-activated systems. Therefore, the coexistence of the broad amorphous halo and sharp diffraction peaks validates the successful formation of a functional, chemically bonded matrix within the recycled geopolymer concrete.

### 3.6. Water absorption

The impact of RBP content and NaOH molarity on water absorption is presented, with results correlated to porosity and mechanical performance. Samples with higher RBP exhibited increased absorption, while 16 M NaOH mixes showed lower rates, confirming their denser matrix and better durability. Water absorption test is a vital indicator of GPC durability and porosity, which in turn influences its strength and longevity.^129,130^ This test assesses GPC's resistance to water ingress, a critical consideration in structural applications prone to water exposure. Research indicates that GPC has superior durability traits, including lower water absorption rates than traditional concrete, thanks to its dense microstructure that impedes water permeability.^131^ Further studies have explored incorporation of industrial by-products, such as fine granite waste powder, into GPC, resulting in enhanced mechanical strength and reduced water absorption.^131^ Activator/binder ratio also plays a crucial role in water absorption; a higher ratio leads to decreased water absorption and fewer permeable voids. Additionally, use of various admixtures and industrial wastes has been investigated to bolster GPC's water resistance, effectively filling concrete matrix's voids, diminishing capillary action, and thus lowering water absorption.^132^ Ongoing research is dedicated to refining GPC's mix design to amplify its water-resistant qualities while simultaneously maintaining or elevating its mechanical prowess. The overarching aim is to develop sustainable, robust, and cost-effective construction materials capable of enduring severe environmental conditions.^133^

As Fig. S2† reveals, average water absorption rates of GRP and GRC samples were analyzed according to ASTM-C1585-20 standard.^114^ Also, 16 M samples have the lowest water absorption rates, with 90 days P-100C-0B-16-2 registering the lowest at 1.8% (Fig. S2a†). The chart shows that higher RBP levels increase water absorption, indicating adding RBP not only reduces strength but also increases water absorption, which is detrimental to GRP durability.

Additionally, molarity plays a significant role in absorption; as molarity decreases, water absorption inversely increases, resulting in P-85C-15B-8-2 having the highest absorption rate at 3.75%. The difference between the highest and lowest water absorption rates is approximately 2%. Therefore, GRP samples with the lowest water absorption rate of 1.8% also exhibit the highest mechanical resistance, and 90 days P-100C-0B-16-2, cured at a temperature of 65 °C, offers the most optimal state in terms of durability and mechanical strength. This is attributed to the fine granularity of the materials, low porosity space, and high structural density within the GRP samples, which prevent water infiltration. Similar to GRP samples, the 16 M GRC samples (Fig. S2b†), were expected to and did have the lowest water absorption rates, and 90 days C-100C-0B-16-2 indeed has the lowest at 4.53%.

The chart further reveals that an increase in RBP correlates with increased water absorption, indicating that adding RBP not only diminishes strength but also raises water absorption, which compromises the durability of GRC. Notably, molarity significantly impacts absorption; as molarity decreases, water absorption inversely increases, culminating in the C-85C-15B-8-2 sample having the highest absorption rate at 6.78%. Generally, the difference between the highest and lowest water absorption rates is about 2.25%, and studies suggest that the rate of water absorption in geopolymer concrete diminishes over time.^134^ Thus, it is inferred that the highest mechanical resistance is achieved with an average absorption rate of 4.53%, and the 90 days C-100C-0B-16-2 sample, cured at 65 °C, represents the most optimal state in terms of durability and mechanical strength. This reflects RA influence and overall porosity on GRC samples' structure. Adding RBP has a detrimental effect on the porosity and water absorption of GRC samples (Fig. S3b†). This increased porosity is due to the introduction of additional interfaces and imperfections within the microstructure. These pathways can facilitate water infiltration, leading to higher water absorption in the GRC network. Conversely, the observed increase in porosity in Fig. S3b† suggests that the geopolymerization reaction may be less effective in the presence of RBP particles, resulting in a less cohesive and more porous structure that provides additional sites for water retention and enhances water absorption.

### 3.7. Density

Density of GRC is a critical factor that influences its mechanical properties and durability. Research indicates that the density of geopolymer concrete (GPC) can vary significantly depending on the composition and the type of aluminosilicate precursors used. Recent studies have explored the impact of various factors on the density of GPC. For instance, the inclusion of NaOH can decrease the density, and the presence of calcium hydroxide can accelerate geopolymerization, leading to an increase in both density and compressive strength, particularly in fly ash-based systems.^135^ Advancements in GPC chemistry and manufacturing techniques have been highlighted, focusing on their influence on the material's physical properties. Using industrial byproducts such as fly ash and GGBS as source materials for GPC contributes to its eco-friendly profile and affects its density.^136^ Moreover, incorporating recycled aggregates in GPC results in a denser, more compact microstructure, which may also affect other physical, mechanical, and durability characteristics.^137^ In summary, the density of GRC is a variable property that is influenced by the raw materials and the manufacturing process. The ongoing research in this field continues to refine our understanding of how to optimize the density of GRC for various construction applications, with a strong emphasis on sustainability and performance.

The study examines impact of density on the compressive strength of GRP samples (Fig. S3†). Within a span of 3 days, the density of the C-100C-0B-16-2 sample increased with the percentage of RBP (Fig. S3d†). Density levels were recorded as 2 g cm^−3^ for C-100C-0B-16-2, 2.11 g cm^−3^ for C-95C-5B-16-2, 2.19 g cm^−3^ for C-90C-10B-16-2, and 2.45 g cm^−3^ for C-85C-15B-16-2. Generally, an increase in RBP percentage led to an increase in density but a decrease in strength, a rule that held true even when molarity was reduced to 12 and 8, as observable in Fig. S3a–c.† Notably, while an increase in density resulted in reduced strength over a period of 3 days, an increase in age of sample to 7, 28, and 90 days was associated with an increase in strength in the mix designs. For instance, the 3 days C-100C-0B-16-2 sample with a density of 2 g cm^−3^ and a compressive strength of 35.34 MPa, compared to the 7 days, 28 days, and 90 days C-100C-0B-16-2 samples, experienced respective increases in density of 2.5%, 5%, and 10%, which directly correlated with an increase in strength. According to Fig. S3a,† RBP absence has a positive effect on the strength and density of GRC samples, resulting in reduced volumetric and apparent density. This decrease in bulk and apparent density is due to the introduction of additional voids and imperfections within the microstructure, which reduces the solid material content per unit volume. Additionally, tightly packed and interconnected matrix observed in Fig. S3a† could potentially contribute to improved compressive strength.

### 3.8. Modulus of elasticity

Modulus of elasticity in geopolymer concrete is a crucial mechanical property that influences its structural behavior and durability. Modulus of elasticity in geopolymer concrete can vary based on different factors such as composition, curing conditions, and presence of fillers like waste marble powder. Geopolymers have shown promising mechanical properties, with compressive strengths exceeding 18 MPa.^138^ The use of pumice powder as a precursor in geopolymer paste has demonstrated the potential to achieve high flexural and compressive strength, especially when cured at 60 °C for 120 hours with an alkali solution of 12 M.^139^ Additionally, prepacked geopolymers containing waste marble powder have exhibited higher compressive and flexural strength when cured at high temperatures, such as 50 °C and 70 °C, compared to ambient temperature curing, showcasing improved mechanical properties and durability for structural applications.^140^

These findings suggest that geopolymer concrete can offer a range of modulus of elasticity values depending on the specific mix design and curing conditions employed. Fig. S4† examines the relationship between the modulus of elasticity, compressive strength, and the percentage of RBP in 90 days GRP samples, in accordance with ASTM-C469/C469M-22 standard. As expected, an increase in compressive strength leads to an increase in modulus of elasticity and *vice versa* (Fig. S4†). Consistent with the mechanical strength results, the C-100C-0B-16-2 sample, with a modulus of elasticity of 32.84 GPa, has the highest value, while the C-85C-15B-8-2 sample has the lowest at 22.13 GPa.

### 3.9. Sustainability analysis

Life Cycle Assessment (LCA) is an evaluation method that analyzes the environmental impacts produced throughout a product's entire lifecycle. LCA addresses environmental aspects and potential impacts, including resource usage and emissions, from the processing of raw materials to production, consumption, recycling, and final disposal—essentially, from cradle to grave.^141^ Today, LCA is recognized as a standard and widely utilized method for the environmental assessment of processes, products, and services. Indeed, LCA acts as the third pillar of a sustainable assessment, complementing technical and economic evaluations, and ensures that, in addition to technical and economic dimensions, actions are environmentally sound.^142^ This study aims to fill existing research gaps in this field in Iran by employing LCA of high-strength concrete and geopolymer concrete. The primary objective is to conduct a comprehensive analysis that provides complete and precise insights into the environmental impact of these concretes' lifecycles. Various institutions and organizations have developed tools and software for LCA to facilitate quick calculations and analysis of large data volumes, driven by the high demand for lifecycle forecasting and environmental outcome analysis of products and services. Consequently, numerous organizations have undertaken to economically justify and enable it for the market and customers. The most popular and widely used LCA software includes Gabi, SimaPro, and OpenLCA. The first two are the oldest tools, and their high cost and closed-source nature have led to increased attention to OpenLCA, which is free and open-source. To achieve the stated goals, OpenLCA software and its free data sources are utilized. The time period for conducting the LCA can affect the results, as energy consumption varies over time.^143^ Additionally, the quality of the assessment results is directly related to the quality of the data used.^144^ Table S1† presents categorization of effects of materials produced and developed with high-strength concretes, and numerical values for each sample.

Percentages of effects of categorization of both materials are compared (Fig. S5†). According to Table S1,† GRC appears to be more environmentally friendly option across most categories, suggesting it could be a more sustainable choice for construction projects concerned with reducing environmental impacts. Numerical values provided in scientific notation (*e.g.*, 6.7 × 10^−10^ for particulate matter) quantify these impacts and allow for a direct comparison between the two materials. Notably, these results are dependent on the quality and scope of data used in LCA.

Fig. S5† presents a comparative analysis of the environmental impacts of HPC and GRC across various categories. In terms of water use, GRC is significantly more efficient, with a value of 20.97, whereas HPC has a recorded impact of 0, suggesting either no impact or a lack of data. Acidification is notably higher for HPC, with a value of 54.09, indicating a greater contribution to acid rain potential compared to GRC. Both materials contribute to nutrient loading in water bodies, but GRC does so to a lesser extent, with a value of 18.87, as opposed to HPC's higher impact of 41.97. When examining climate change, GRC shows lower impacts in subcategories such as climate change-biogenic and climate change-fossil fuel & cement process emissions combined, with values of 0.52 and 25.89, respectively, compared to HPC's higher impacts. The ozone depletion potential of HPC is concerning at a value of 4.30, especially in light of global efforts to reduce harmful emissions. GRC's lower energy resource use, scored at 14.47, suggests it is a more sustainable building material. In terms of toxicity, GRC has lower human toxicity (non-cancer effects) and ecotoxicity impacts, with values of 25.47 and 66.85, respectively, indicating a reduced environmental burden. Lastly, land use impacts are present for both materials, with GRC's impact quantified at 11.00, likely reflecting the land needed for raw material extraction or processing. Overall, GRC appears to be the more environmentally friendly option in most categories.

Overall, Fig. S5† suggests that GRC generally has a lower environmental impact across most categories compared to HPC, making it an eco-friendlier option in construction. Particularly, results' quality is directly related to the data quality used in the LCA. The assessment's time period also plays a role, as longer periods may result in changes in energy consumption and other variables affecting the results. This analysis can inform decision-making in construction projects, especially when prioritizing environmental sustainability, it is also a valuable tool for researchers addressing the environmental challenges associated with construction materials.

In addition to the parameters examined in this work, several other factors can be optimized to further enhance the performance of geopolymer recycled concrete. These include adjusting curing temperature and duration to promote favorable microstructural evolution, modifying Si/Al and Na/Al ratios to improve gel formation, and exploring the use of alternative activator systems such as potassium-based solutions. The incorporation of nano-scale or fiber reinforcements (*e.g.*, nano-silica, basalt, or polypropylene fibers) may further improve ductility, crack resistance, and long-term durability. Moreover, surface treatment of recycled aggregates could enhance interfacial bonding and reduce porosity, contributing to overall performance gains. These considerations are proposed as directions for future research to expand the practical applications of this sustainable material system.

## 4. Conclusions

This study presents a novel and sustainable approach to fully recycling ordinary Portland cement (OPC) concrete by utilizing it as both recycled aggregate (RA) and recycled concrete powder (RCP), in combination with recycled brick powder (RBP) as an aluminosilicate additive in geopolymer systems. Through alkaline activation using sodium hydroxide and water glass, a series of geopolymer recycled concretes (GRC) and pastes (GRP) were successfully synthesized and evaluated.

The research significantly advances the current state of knowledge by demonstrating that:

- A fully recycled geopolymer concrete can be produced without relying on virgin materials or energy-intensive re-clinkering processes.

- Incorporating RA and RCP improves long-term mechanical strength, achieving up to 90% compressive strength gains at 90 days compared to GRP alone.

- While the addition of RBP reduces mechanical strength and modulus, it enhances flowability, aesthetics, and crack resistance, offering a functional trade-off depending on application priorities.

- Microstructural analysis confirmed enhanced adhesion within the geopolymer matrix due to the chemical similarity between RCP and RA.

- A comprehensive Life Cycle Assessment (LCA) verified the environmental superiority of GRC over conventional OPC concrete in terms of emissions, resource use, and toxicity.

In conclusion, these findings support the classification of GRC as a high-performance structural material and demonstrate its strong potential for sustainable construction applications. This study contributes to the development of circular economy strategies in the construction sector by valorizing demolition waste and reducing the carbon footprint of concrete production. One key limitation of this study is the inherent variability in the quality of waste concrete, including factors such as cement type, hydration degree, admixtures, and aggregate characteristics. These variables can influence the reactivity of recycled concrete powder (RCP) and the bonding behavior of recycled aggregates (RA), thereby affecting mechanical performance. Although materials were characterized using EDS and XRD and processed through standardized methods, complete uniformity cannot be guaranteed. As such, results should be calibrated against locally sourced materials to ensure reproducibility across different contexts.

While the present study focused on evaluating the mechanical performance of geopolymer recycled concrete (GRC) up to 90 days, which aligns with commonly accepted benchmarks in geopolymer research, it does not encompass long-term durability aspects. Extended testing—such as carbonation resistance, freeze-thaw cycling, chloride ingress, and sulfate attack—remains essential to assess the long-term behavior of GRC under environmental exposure. Future investigations will address these parameters to comprehensively evaluate the structural applicability, durability, and service life of GRC in practical construction scenarios.

## Conflicts of interest

Authors declare that they have no known conflict of interests or personal relationships that could have appeared to influence the work reported in this paper.

## Supplementary Material

RA-015-D5RA02249E-s001

## Data Availability

The data associated with this work will be made available on request.
