# Supplementary material for: Toward high sustainability using fully recycled geopolymer concrete: mechanical, rheological, and microstructural properties
Source: RSC Adv. 2025 Jul 4;15(28):22953–71. doi: 10.1039/d5ra02249e (PMC12226849; doi:10.1039/d5ra02249e)
Supplement: RA-015-D5RA02249E-s001 [file RA-015-D5RA02249E-s001.pdf]

Supplementary Information (SI) for RSC Advances.
This journal is © The Royal Society of Chemistry 2025

Toward high sustainability using fully recycled geopolymer concrete: Mechanical, rheological, and microstructural properties

Faramarz Moodi[a,*], Mohammad Reza Hanafi[a] , Zahra Shariatinia[b,*]

[a]Department of Civil and Environmental Engineering, Amirkabir University of Technology, Tehran, Iran

[b]Department of Chemistry, Amirkabir University of Technology, Tehran, Iran

[*] Corresponding authors.
E-mail addresses: fmoodi@aut.ac.ir (F. Moodi), shariati@aut.ac.ir (Z. Shariatinia)

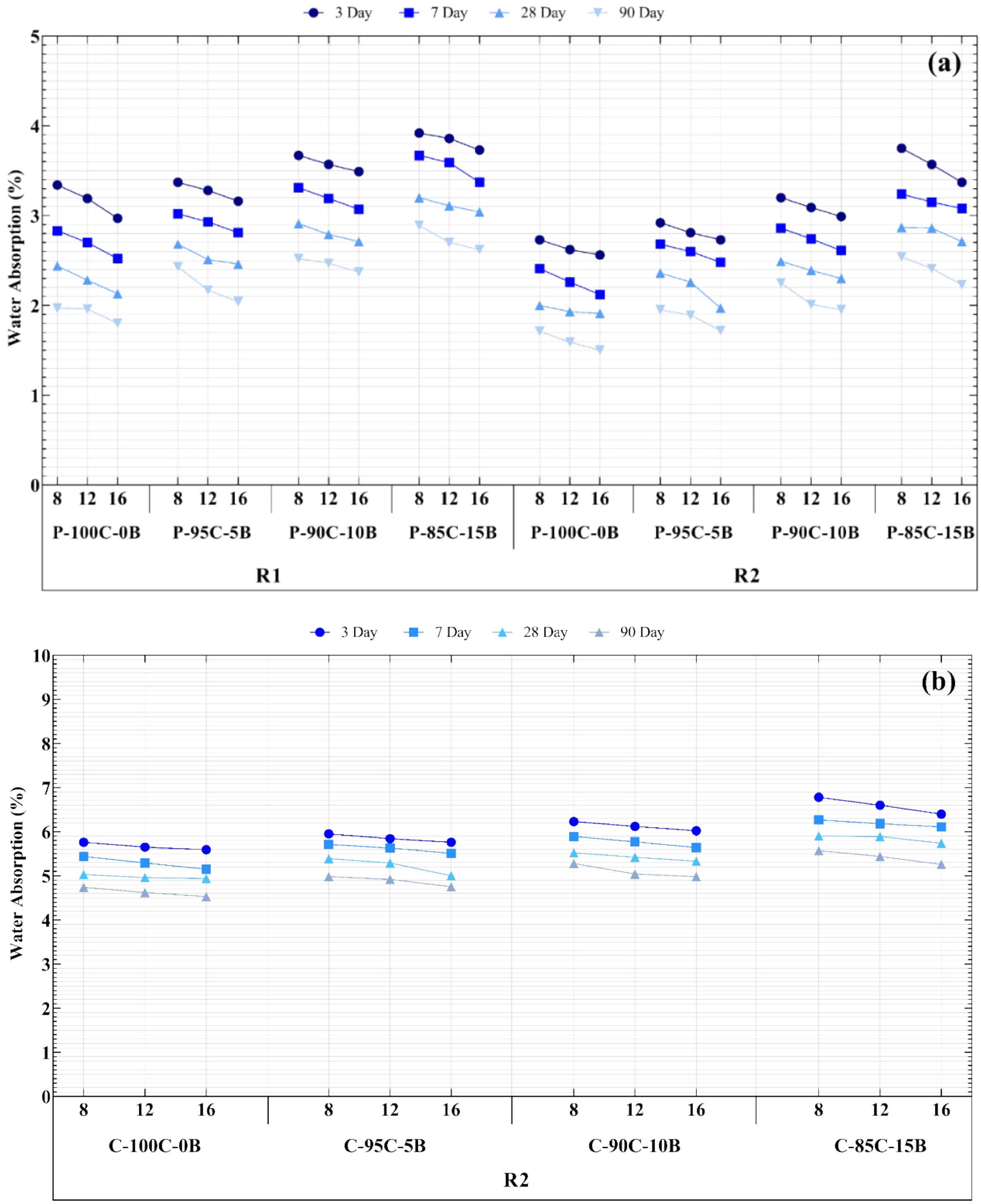

Fig. S1. The average percentage of water absorption of a) GRP samples, and b) GRC samples.

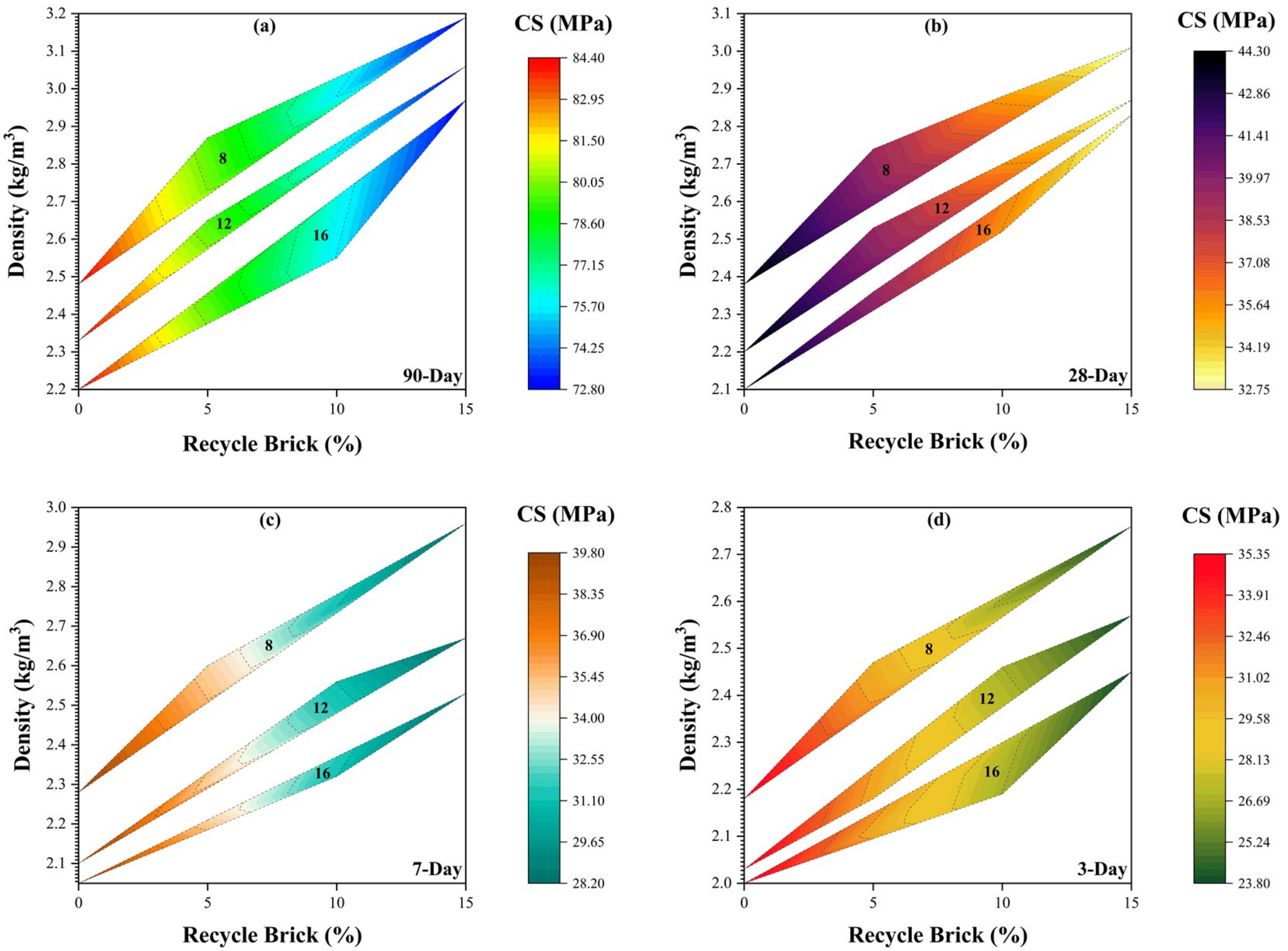

Fig. S2. Investigating the effect of RBP percentage, and density on compressive strength GRC of samples in: a) 90-day, b) 28-day, c) 7-day, and d) 3-day.

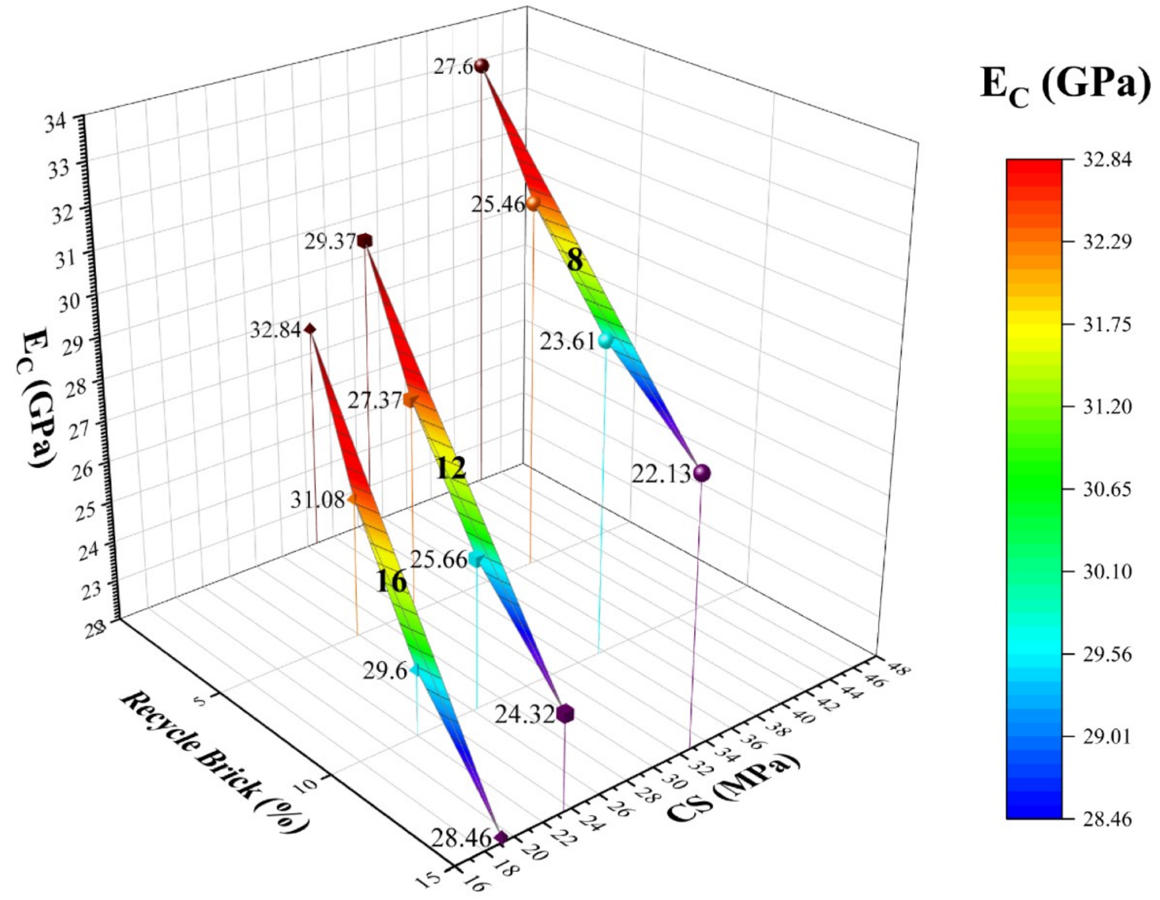

Fig. S3. Investigating the effect of RBP percentage, and Modulus of elasticity on compressive strength of GRC samples in 90 days.

Table S1. Impact category according to the GRC and HPC samples.

Impact category	Reference unit	GRC	HPC
Particulate Matter	disease inc.	6.7×10^{-10}	1.4×10^{-9}
Resource Use, Minerals and Metals	kg Sb eq	3.4×10^{-9}	5.0×10^{-9}
Human Toxicity, Cancer	CTUh	1.4×10^{-11}	9.1×10^{-11}
Eutrophication Marine	kg N eq	1.1×10^{-5}	6.2×10^{-5}
Climate Change-Fossil	kg CO_2 eq	2.4×10^{-2}	9.1×10^{-2}
Land Use	Pt	1.1×10^{-3}	0.0
Ecotoxicity, Freshwater	CTUe	1.8×10^{-2}	2.7×10^{-3}
Eutrophication, Freshwater	kg P eq	4.0×10^{-8}	9.4×10^{-8}
Ionising radiation, Human Health	kBq U-235 eq	1.2×10^{-5}	1.1×10^{3}
Climate Change	kg CO_2 eq	2.4×10^{-2}	9.1×10^{-2}
Ozone Depletion	kg CFC11 eq	1.4×10^{-14}	3.2×10^{-13}
Resource Use, Fossils	MJ	1.3×10^{-1}	3.8×10^{-1}
Climate Change-Land Use and Land Use Change	kg CO2 eq	9.4×10^{-7}	5.9×10^{-5}
Human Toxicity, Non-Cancer	CTUh	1.2×10^{-9}	4.6×10^{-9}
Photochemical Ozone Formation-Human Health	kg NMVOC eq	6.5×10^{-5}	1.7×10^{-4}
Climate Change-Biogenic	kg CO_2 eq	2.9×10^{-7}	5.5×10^{-5}
Eutrophication, Terrestrial	mol N eq	1.3×10^{-4}	6.7×10^{-4}
Acidification	mol H+ eq	1.1×10^{-4}	1.9×10^{-4}
Water Use	m^3 depriv.	5.5×10^{-4}	1.5×10^{-2}

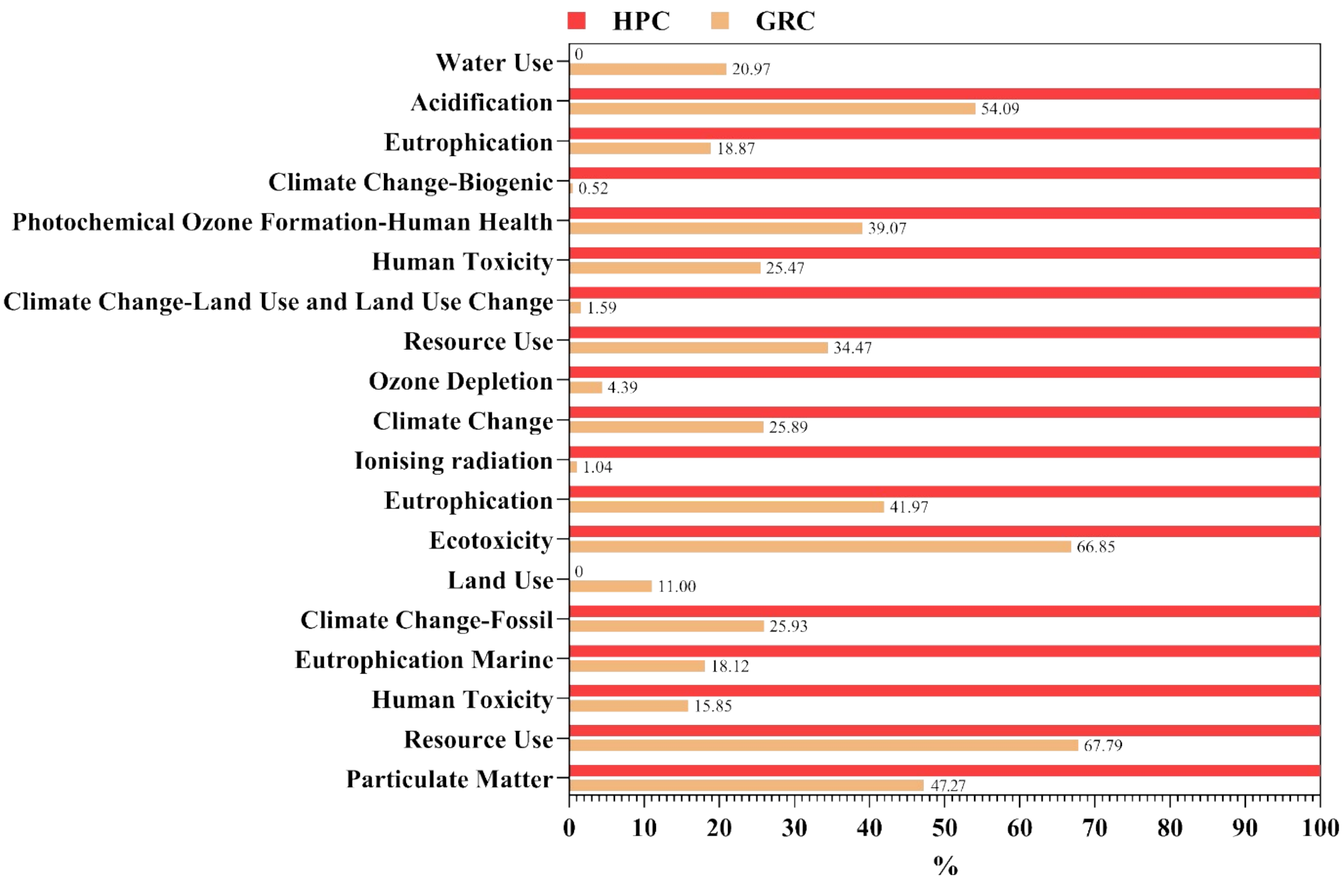

Fig. S4. Comparison of Impact category related to GRC and HPC samples.
